# A Simple Method for Removing Foreign Bodies from the Nasal Cavities of Children

**Published:** 1911-02

**Authors:** 


### A Simple Method For Removing Foreign Bodies From the Nasal Cavities of Children.

According to Dr. G. Bieser, the employment of the usual methods for removing foreign bodies from the nasal cavities in struggling children and without anesthesia is attended not only with the dangers from traumatism, but also with difficulty and occasional failure. The employment of serodynamics may overcome these objections. The method advised by the author is as follows: The child is placed in the ordinary position for intubation, the assistant holding his hand snugly over the child's mouth; one end of a piece of rubber tubing is snugly inserted in the nostrils opposite the one holding the foreign body, the other end is inserted into the operator's mouth; the operator then blows suddenly and vigorously into the nostril and dislodges the offending body. The simplicity, cleanliness and efficiency of this method are apparent, the child's struggles causing no traumatism. —*Southern California Practitioner.*

---

That bane of the boarding house, the bedbug (cimex lectularius) is to be banished from the commonwealth of Wisconsin forthwith, since the Wisconsin Medical Women's Association, in annual convention assembled, has passed resolutions condemning the insect. The president of the association in her annual address declared her conviction that the parasite mentioned is one of the greatest enemies of the public health, and is one of the chief factors in the conveyance of syphilis in obscure cases. She asked, "What could be more plausible than that persons, traveling about, occupying a bed that had been previously used by hundreds of other persons, would be infected by the bedbug, which had feasted on all kinds of unclean creatures?" While this is a new suggestion, it is, of course, plausible. We know that the germs of anthrax have been conveyed by the insect. The bacillus lepræ has been harbored by it, as also the plasmodium malariæ and the comma bacillus. It is more than suspected that it is the host of the plague bacillus, and many severe exanthematous diseases may be widely distributed by the nasty, rusty, disgusting insect. The work of the Wisconsin Medical Women's Association in fighting all germ carriers, including the cimex lectularius, is to be commended.—*Lancet-Clinic.*

